# Revealing the different levels of action monitoring in visuomotor transformation task: Evidence from decomposition of cortical potentials

**DOI:** 10.1111/psyp.14708

**Published:** 2024-10-14

**Authors:** Nikolay Syrov, Daha Garba Muhammad, Alexandra Medvedeva, Lev Yakovlev, Alexander Kaplan, Mikhail Lebedev

**Affiliations:** ^1^ Vladimir Zelman Center for Neurobiology and Brain Rehabilitation Skolkovo Institute of Science and Technology Moscow Russia; ^2^ Laboratory for Neurophysiology and Neuro‐Computer Interfaces, Department of Human and Animal Physiology, Faculty of Biology Lomonosov Moscow State University Moscow Russia; ^3^ Faculty of Mechanics and Mathematics Lomonosov Moscow State University Moscow Russia; ^4^ Sechenov Institute of Evolutionary Physiology and Biochemistry Russian Academy of Sciences St. Petersburg Russia

**Keywords:** multivariate pattern analysis, P3, reafferent potential, residue iteration decomposition, response monitoring, theory of event coding, visuomotor transformation

## Abstract

This study investigates the cortical correlates of motor response control and monitoring, using the Theory of Event Coding (TEC) as a framework to investigate signals related to low‐level sensory processing of motor reafference and high‐level response monitoring, including verification of response outcomes with the internal model. We used a visuomotor paradigm with two targets at different distances from the participant. For the recorded movement‐related cortical potentials (MRCPs), we analyzed their different components and assessed the movement phases during which they are active. Residual iteration decomposition (RIDE) and multivariate pattern analysis (MVPA) were used for this analysis. Using RIDE, we separated MRCPs into signals related to different parallel processes of visuomotor transformation: stimulus processing (S‐cluster), motor response preparation and execution (R‐cluster), and intermediate processes (C‐cluster). We revealed sequential activation in the R‐cluster, with execution‐related negative components and positive contralateral peaks reflecting reafference processing. We also identified the motor post‐imperative negative variation within the R‐cluster, highlighting the response outcome evaluation process included in the *action file*. Our findings extend the understanding of C‐cluster signals, typically associated with stimulus–response mapping, by demonstrating C‐activation from the preparatory stages through to response termination, highlighting its participation in action monitoring. In addition, we highlighted the ability of MVPA to identify movement‐related attribute encoding: where statistical analysis showed independence of stimulus processing activity from movement distance, MVPA revealed distance‐related differences in the S‐cluster within a time window aligned with the lateralized readiness potential (LRP). This highlights the importance of integrating RIDE and MVPA to uncover the intricate neural dynamics of motor control, sensory integration, and response monitoring.

## Abstract 2

Our findings identified components, including the LRP, reafference potential (RAP), and motor post‐imperative negative variation within the same study. Moreover, we revealed several RAPs occurring at critical response moments, highlighting the role of this sensory processing in response control.

## 1 INTRODUCTION

While sensorimotor integration is the umbrella term that encompasses the processes of coordinating sensory information into the appropriate voluntary movement, visuomotor transformation encompasses how the visual stimulus results in motor action. As discussed by the Theory of Event Coding (TEC), this process is a three‐step mechanism involving three files called *object*, *event*, and *action* files (Hommel et al., 2001). The information about the stimulus such as the position of the target, its size, shape, and other features are stored in the *object file*. The details of the movement preparing to reach the target, such as the number of joints, are contained in the *action file*, while the *event file* provides the link between the *object* and *action files* (Hommel et al., 2001).

Although the sensory inputs are encoded in the *object file* (Hommel et al., 2001), external target‐related signals modulate the kinematic settings specified in the *action file*. During movement execution, continuous sensory feedback, both internal and external, contributes to online adjustments and updates the motor plan for subsequent trials. Accurate integration of sensory information is crucial for goal‐directed movements. This integration should occur both before movement and throughout its execution (Edwards et al., 2019).

To track such rapidly evolving processes before and during movement, motor‐related cortical potentials (MRCPs) recorded with electroencephalography (EEG) can be useful because they allow the study of cortical activity with high temporal resolution (Shakeel et al., 2015). Several factors such as velocity and force of movement were shown to be reflected in MRCP changes (Nascimento et al., 2006; Syrov et al., 2024). The morphology of MRCPs is modulated not only by *action files* but also by stimulus attributes conveying target‐related information. Specifically, visual properties such as target size and distance have been demonstrated to influence the activation of sensorimotor cortices, thereby shaping the MRCP components (Kourtis et al., 2012; Kirsch et al. (2010), Ifft et al., 2011; De Sanctis et al., 2013).

Concurrently, the MRCP components underlying action monitoring and outcome evaluation have received comparatively less empirical attention. However, the processing of incoming sensorimotor feedback during movement execution, as well as the analysis of action results with respect to goal attainment, represents a critical neurophysiological process. Currently, the role of action monitoring is primarily attributed to several potentials. Prominent candidates involved in this process are the P3 family of event‐related potentials and the late positive complex (LPC) originating from frontoparietal networks. These potentials shown to index *event* *file* activity serve crucial functions in stimulus–response (S–R) mapping and updating of internal environmental models based on novel sensory input, thereby modulating expectations about upcoming events (Donchin & Coles, 1988, Brydges & Barceló, 2018; Geng & Vossel, 2013; Verleger et al., 2005, Husain & Nachev, 2007; Corbetta et al., 2008; Eickhoff et al., 2011, Geng & Mangun, 2011,Langner & Eickhoff, 2013, Seghier, 2013). Another potential identified is motor post‐imperative negative variation (mPINV), a negative post‐movement deflection with sensorimotor sources. This deflection is thought to reflect an evaluation of response efficacy (Bender et al., 2006, 2012). Complementary to high‐level cognitive processing, low‐level processing of afferent signals is manifested in another MRCP component known as reafferent potential (RAP) (Berchicci et al., 2016; Bötzel et al., 1997; Shibasaki et al., 1980; Syrov et al., 2024). While P3 is often conceptualized as a component of the *event file*, RAP and mPINV are considered to be related to *action* files (Bender et al., 2006; Syrov et al., 2024). In the current literature, however, this distinction has not been made explicitly. Furthermore, these components have not been described simultaneously within a single experimental paradigm. This absence raises questions about the specificity of these potentials to particular experimental conditions rather than to general movement performance monitoring. For example, similarities in polarity, latency, and topography between P3 and RAP in S–R tasks (SRT) may complicate their distinction. Regarding the discussion in (Verleger et al., 2005) the nature of P3 and RAP may actually be mixed, since the monitoring process reflected in P3 must also include analysis of somatosensory afferent signaling the onset of the intended action. Moreover, some studies describe reafferent activity as P3 based on latency, but not on hypothesized functions, which adds ambiguity to the issue (Shibasaki et al., 1980). Therefore, the aim of the present study was to describe in detail the different neural components associated with action execution and monitoring, which are fundamental aspects of TEC, in the SRT paradigm. By examining the temporal dynamics and spatial distribution of these components, we aimed to distinguish sensorimotor and cognitive levels of response monitoring and action outcome evaluation.

We used a visuomotor task with two buttons positioned at different distances from the participant—one in close proximity and the other up to 40 cm away. This design allowed us to study conditions that varied in movement duration, with a clear temporal separation between movement initiation and goal attainment (button press). We used advanced methods such as the Residue Iteration Decomposition (RIDE) algorithm (Ouyang et al., 2011) and multivariate pattern analysis (MVPA) to distinguish brain signal components associated with different neural processes, namely stimulus processing (S‐cluster), motor response preparation and execution (R‐cluster), and intermediate linking processes (C‐cluster), and to analyze their temporal dynamics within a visuomotor task with different target distances. A clear delineation of the functional role of components associated with cognitive control and context updating, such as P3/LPC, as well as sensorimotor potentials, such as RAP and mPINV, can provide critical insight into how the brain at different levels integrates sensory inputs during continuous execution to perform accurate voluntary actions according to the intended motor plan. Given the well‐established role of the P3 in linking sensory input to corresponding responses (Ouyang et al., 2017; Petruo et al., 2017; Verleger et al., 2014; Wolff et al., 2017), we expected RIDE to assign the P3 and fronto‐parietal P3‐like components to C‐cluster. In line with TEC, we predicted that MVPA would reveal the temporal stability of C‐cluster activation, encompassing both premotor S–R mapping and further execution phases, as its signals reflect continuous action monitoring over the entire duration of the movement. Conversely, we expected RAP and mPINV to be exclusive to the R‐cluster, strictly related to motor execution activity and somatosensory feedback processing, and correlated with reaction times.

## 2 METHODS

### 2.1 Participants

This study involved 20 right‐handed individuals (6 females and 14 males) with no history of neurological disorders and normal or corrected‐to‐normal vision. Subjects were 21–34 years old (mean age **±** std: 25.39 **±** 3.03). The study protocol was developed and conducted in accordance with the *Declaration of Helsinki* and approved by the local ethics committee of the Skolkovo Institute of Science and Technology (Protocol #10, dated 18.05.2023). Informed written consent was obtained from all participants prior to enrolling them in the study. Participants were fully informed of the objectives of the study and were free to inquire or withdraw at any time during the research process.

### 2.2 Study design and procedures

**FIGURE 1:**
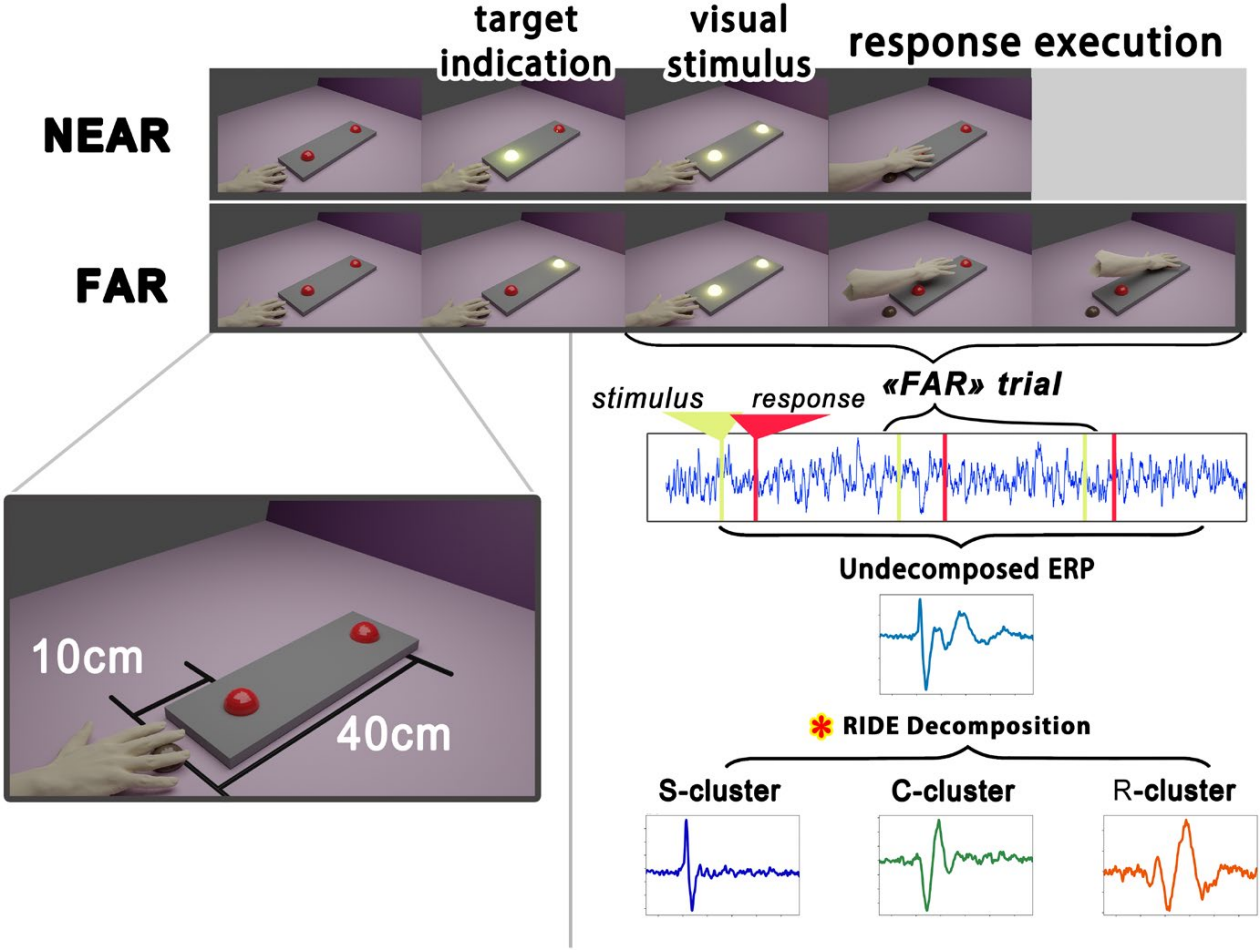
The experimental design and data processing pipeline. Two linearly distanced target buttons were to be reached and pressed in response to flashlights (*far* and *near*). The participant's right hand rested on the start button from which each response was initiated and terminated. Each block of trials began with the identification of the target button, after which each flashlight required reaching and pressing the target button. A total of 6 *far* and 6 *near* blocks of trials were performed, resulting in 180 responses per condition per subject. EEG signal recording was synchronized with the presentation of the visual stimuli and the button presses. Response times in each trial were used for EEG signal decomposition with the RIDE algorithm. Stimulus‐related (S), response‐related (R), and intermediate signals (C) were decomposed in separate clusters. An example of decomposed MRCP and three clusters obtained with RIDE are shown in the lower right panel. The red asterisk connects the RIDE decomposition scheme with the data processin g pipeline shown in Figure 2.

Participants sat in front of a panel with three buttons at varying distances from each other. The right hand rested on the *start* button, which was positioned ~15 cm from the participant. The other two buttons had to be pressed: the *near* button was placed 10 cm and the *far* button 40 cm from the *start* button (Figure 1). In response to visual cues (button flashes), participants reached toward one of the buttons, pressed it, and then returned their hand to the start. Although both targets flashed simultaneously, they were instructed to press only the designated target, which was indicated by four rapid flashes before the start of each block of trials. Each block of trials consisted of 15 trials with a flash duration of 100 ms and interstimulus intervals randomized between 700 and 2000 ms. The experimental session consisted of 12 blocks of trials, equally divided between *near* and *far* conditions and split into two separate 6‐block runs to minimize fatigue. The order of the *near* and *far* blocks of trials was randomized. Thus, an example of an experimental session can be represented by the sequences FNNFNF + FNFFNN, where F and N represent *far* and *near* button blocks of trials correspondingly. This resulted in a total of 180 trials per target for each participant.

### 2.3 Signal acquisition and processing

Signal acquisition was performed using an NVX‐36 amplifier (MCS, Russia) and 30 EEG *Ag*/*AgCl* gel‐based passive electrodes placed on the scalp according to the international “10/10” system in the following positions: *Fp1, Fp2, Fz, FC1, FC2, FC3, FCz, FC4, FC5, FC6, C5, C3, Cz, C4, C6, CP1, CP2, CP3, CPz, CP4, CP5, CP6, P3, Pz, P4, PO3, POz, PO4, O1, O2* with *A1* and *A2* as references. The electrode‐skin impedance was kept below 20 kΩ. In addition, two electromyography (EMG) electrodes monitored muscle activity on the right *m. biceps brachii*. Signals were sampled at 500 Hz. The signal acquisition was synchronized with the presentation of the visual stimuli and the key presses with an analogous triggering (without delay).

**FIGURE 2:**
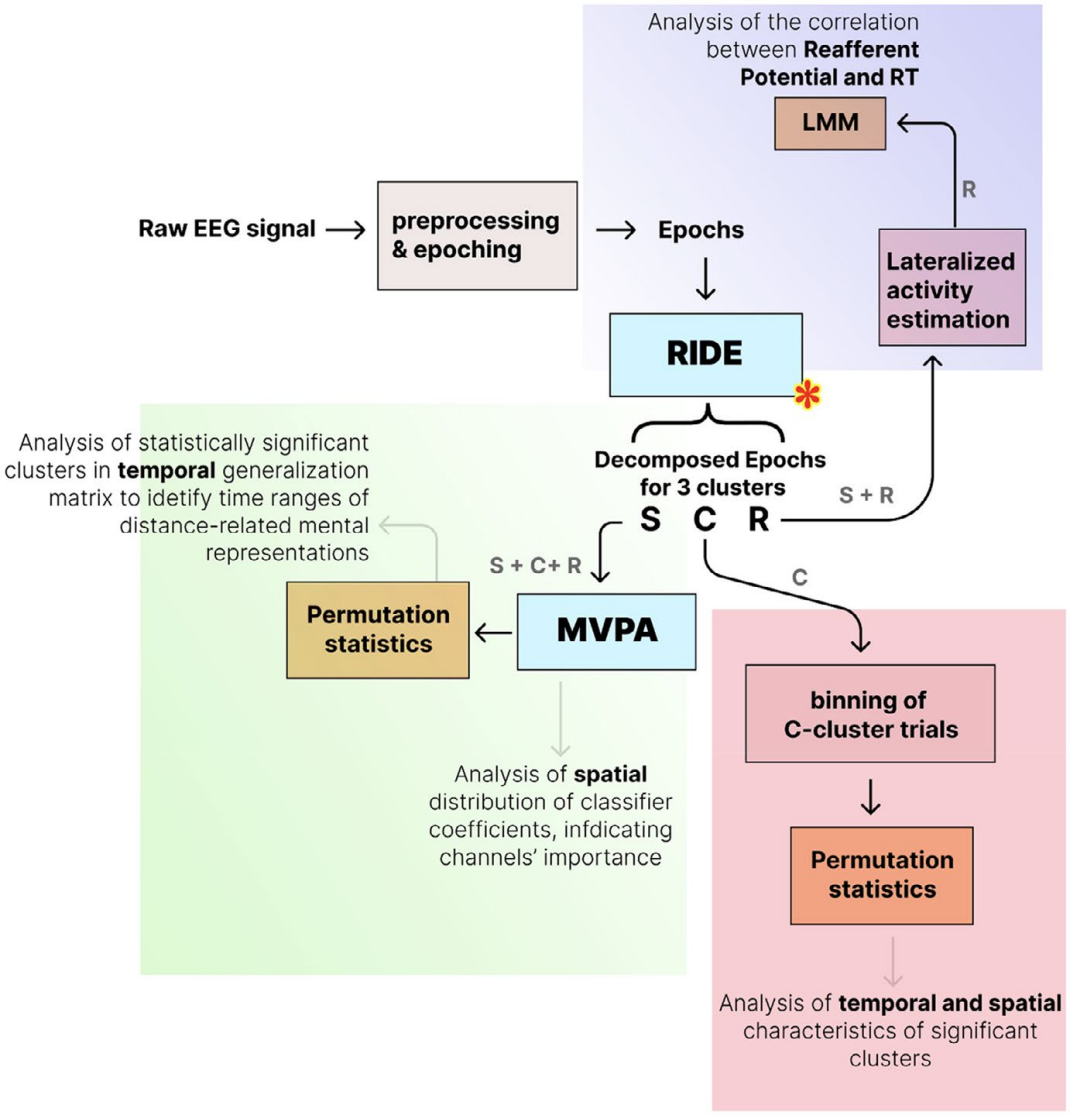
The conceptual scheme of data processing and analysis. The red asterisk shows the place of the RIDE decomposition within a processing pipeline (see also Figure 1). Letters S, C, and R refer to RIDE clusters. The letters near the arrows indicate which cluster data was used in the corresponding analysis step.

Figure 2 shows the pipeline of the methodological approaches employed in this study. It delineates the sequential stages of signal preprocessing, decomposition algorithms, and statistical analyses implemented. EEG preprocessing included visual inspection of the data to identify and interpolate bad channels, and deletion of epochs in which artifacts were detected. Bad channel interpolation was performed using the spherical spline method (Perrin et al., 1987). The number of interpolated channels varied for each participant and did not exceed 10%. Epochs affected by large amplitude drifts exceeding approximately **±**40 μV were visually inspected and manually deleted. On average, 2 ± 1 epochs were deleted per participant. The signal was then filtered in the 0.1–30 Hz frequency range using a zero‐phase FIR filter with a Hamming window, and independent component analysis (*fastICA*) was performed to remove artifacts related to electrooculogram and muscle activity. We used visual inspection of ICA sources to remove the components related to horizontal eye movement and muscle artifact. All preprocessing steps were performed using MNE‐Python v1.3.1 (Gramfort et al., 2013).

For MRCP analysis, a 1–15 Hz band‐pass filter was used to remove high‐frequency oscillations that could contaminate the shape of the evoked responses. The signal was then segmented into −0.5 to 2 s epochs from the onset of the visual stimulus. For baseline correction, the interval from −0.2 to 0 s before the stimulus was used.

### 2.4 Analysis of behavioral data

Response time was defined as the interval between the flash onset and the moment of the target button press for each trial. Movement onset was identified by detecting an increase in the EMG signal, specifically when the root mean square (RMS) transformed EMG exceeded two standard deviations above the baseline. These time markers were then used to isolate different MRCP components using RIDE. Movement duration was defined as the time from movement onset to the target button press.

### 2.5 Signal decomposition with RIDE

To analyze MRCP components associated with different neural processes, we used the RIDE decomposition algorithm, which has been shown in previous studies to be a useful tool for elucidating sensorimotor integration (Ouyang et al., 2013). The RIDE separates brain signals into three clusters: *stimulus‐locked* (S), *response‐locked* (R), and *an intermediate cluster* (C). The C‐cluster signals are characterized by significant latency variability and are not directly locked to stimulus onset or response time. The RIDE assumes that these clusters are linearly combined within each EEG trial and can be separated through iterative analysis of the ERPs obtained with stimulus‐ and response‐locked averaging and the residuals, which are the result of a subtraction of the averaged ERP from the single‐trial signals. A comprehensive review of the principles and applications of the RIDE method can be found in Ouyang et al. (2011), Stürmer et al. (2013), and Ouyang et al. (2015a).

For better separation and convergence of the decomposition, RIDE requires predefined time windows in which to find the activity associated with each cluster. For our analysis, the S‐cluster was analyzed within a window of 0–600 ms, the C‐cluster within 100–700 ms, and the R‐cluster from −500 to 700 ms. Notably, the anchor time moment for the R‐cluster is the moment of the target button press in each trial, whereas the time windows for the S‐ and C‐clusters are locked to the onset of the stimulus. The selection of these time windows was guided by previous studies (Ouyang et al., 2015a) and fine‐tuned according to our decomposition results. Specifically, for the R‐cluster, we evaluated the effectiveness of the decomposition using both movement onset (identified by EMG) and RT (the moment the target button was pressed). The results demonstrated that the use of RT for the decomposition resulted in more consistent R‐cluster separation, effectively preventing the mixing of C‐ and R‐clusters. See Appendix S1 for a detailed comparison.

The decomposition procedure was performed individually for each participant and condition using the RIDE toolbox (version as of 16/01/2016) for Matlab (MathWorks, USA) with a pipeline described in (Ouyang et al., 2015b).

### 2.6 Analysis of decomposed signals

#### 2.6.1 Lateralized activity estimation

To estimate motor‐related lateralization in cortical activity Coles (1989) proposed a double subtraction method. In this method, the signal from the *C4* channel is subtracted from the signal from the *C3* channel for right‐handed responses, and vice versa for left‐handed responses. After, the results are averaged to obtain a general motor‐related lateralized response. However, due to the lack of left‐handed responses in our study design, we used a single subtraction (*C3–C4*) instead of the traditional approach. Lateralization was analyzed in S‐ and R‐cluster signals, as both clusters have been previously identified to exhibit LRP (Stürmer et al., 2013).

#### 2.6.2 Analysis of R‐cluster signals

We expected to detect lateralized RAP‐related activity within the R‐cluster and expected to find a correlation between RT and RAP latency. RAP latency was defined as the time of the maximal positive value within the time interval from the button press to 200 ms after. Since the signal was filtered in the 1–15 Hz band and only response‐related activity was isolated, single‐trial R‐signals were not significantly contaminated by noise. This enhanced the reliability of determining single‐trial latency; however, latency estimation in individual trials is still prone to noise. To mitigate noise effects, we combined all trials for all participants, thereby increasing the number of samples for correlation. Thus, to investigate the relationship between R‐cluster peaks and RTs at the single‐trial level, a linear mixed model (LMM) analysis was performed. With this approach, we accounted for the inter‐ and intrasubject variability by considering all trials instead of averaging within subjects (Brauer & Curtin, 2018). RT served as the dependent variable, RAP latency as the predictor, and subject variability was included as a random effect to account for both inter‐ and intra‐subject variability without averaging data within subjects.

To better illustrate the relationships between R‐cluster components and button pressing time, we collected all R‐trials from the *C3* channel in a matrix, sorted them in ascending order by RT, and applied a moving average with a 10‐ms window and a timestamp step to each epoch, resulting in smooth traces (Figure 4b).

#### 2.6.3 Analysis of C‐cluster signals

Movement durations were used in the analysis of C‐cluster signals, where it was expected that monitoring process correlates would be encoded. Thus, we expected that trials with longer movements would be characterized by changes in C‐cluster signals compared to trials with shorter movements. Movement duration, an important but often neglected metric, was highlighted by Courchesne (1978) for its critical role in capturing activity associated with controlling and monitoring execution. Using a binning approach similar to Roth et al. (1978) and Poli et al. (2010), we selected C‐trials based on the extremes of movement duration. Trials were ordered by duration, and averages were computed for different quartiles, separating the shortest (Q1) from the longest (Q4) responses. For each subject, two subsets of C‐cluster signals were taken for further statistical analysis. For visualization, the C‐cluster signals from the *Pz* channel were aggregated in the order of increasing duration of the movement and smoothed with a moving average of 10 ms (see Figure 5a).

To compare C‐trials with the shortest and longest responses, we used a spatio‐temporal cluster‐level statistical permutation test using a threshold‐free approach (Maris & Oostenveld, 2007; Sassenhagen & Draschkow, 2019). By generating 100,000 random permutation sets, we established a significance level of *p*‐value = 0.01. We used permutation tests to determine the overall differences between the C‐cluster signals in the shortest and longest responses. We did not use it to make unwarranted inferences about the precise timing or spatial location of these differences. Instead, we focused on matching the cluster characteristics with the MRCP components being studied.

### 2.7 Multivariate pattern analysis results

#### 2.7.1 Rationale for MVPA and basic principles

We used MVPA to gain insight into the temporal organization of the RIDE clusters and to define the response stages at which target distance is encoded in brain signals. Originally used in functional magnetic resonance imaging studies, MVPA has recently been recommended for EEG analysis (King & Dehaene, 2014). Many EEG studies have reported that MVPA is an effective tool for mapping the temporal evolution of mental representations. Moreover, MVPA may be more sensitive than statistical approaches and provides a more reliable estimation of the temporal windows of effect exposition (Fahrenfort et al., 2018; Grootswagers et al., 2017; Li et al., 2022; Mückschel et al., 2017; Petruo et al., 2021; Takacs et al., 2021a; Takács et al., 2022).

When applying MVPA to EEG data, the methodology involves the training of a classifier with the use of data from a single time point (the number of features equals the number of sensors). In the second step, the predictive accuracy of the model is evaluated on trials from the test sample. The same time moment is used.

To evaluate a model's ability to generalize—determining whether the neural code identified at time *t* reappears at later times *t'*—the prediction score is assessed on test trials using data from other time points. Applying a model across different time points results in a temporal generalization matrix with performance scores that indicate whether the specific neural code remains stable or evolves dynamically. This matrix, with rows representing training times and columns representing test times, highlights the temporal dynamics of neural coding (see King & Dehaene, 2014 for further details).

#### 2.7.2 Implementation of MVPA

We applied MVPA on decomposed data by initially extracting single‐trial signals for the S, C, and R‐clusters using the *move3* function from the RIDE toolbox (Takács et al., 2022). The same MVPA pipeline was applied to the data from each cluster independently.

Using Fisher's linear discriminant analysis from the ScikitLearn v1.5.0 (Pedregosa et al., 2011), we trained the model to discriminate between *far* and *near* trials. We split the trials into training and validation sets, allocating 80% of the trials to training and 20% to validation. This process was repeated five times for each subject, with trials randomly assigned to the training and validation sets in each iteration. Decoding accuracy was assessed on each validation iteration using the area under the curve (AUC) metric (Fahrenfort et al., 2018).

#### 2.7.3 Analysis of MVPA results

Thus, after the previous steps, we obtained five generalization matrices for each subject, one from each validating iteration, which were then averaged. The averaged matrices from all subjects were used to identify temporal patterns of statistically significant decoding between specific cluster signals in *far* and *near* trials. We used a cluster‐level statistical permutation test. It was expected that the AUC values would be statistically above the chance level of 0.5 within the time intervals in which distance‐related information is encoded. Thus, we compared square generalization matrices, containing the AUC values, with matrices of the same size filled with the value 0.5. We set the *F*‐threshold at 8.0 and the significance level at a *p*‐value of 0.01.

After we aimed to identify which EEG channels contributed most to the classification of *near* and *far* trials. To achieve this, the model coefficients were analyzed for time intervals where the decoding performance was the highest. Channels with higher coefficients contributed more significantly to distinguishing between *near* and *far* trials. The coefficients were visualized using topographic maps.

## 3 RESULTS

### 3.1 Analysis of undecomposed MRCPs and RIDE clusters

**FIGURE 3:**
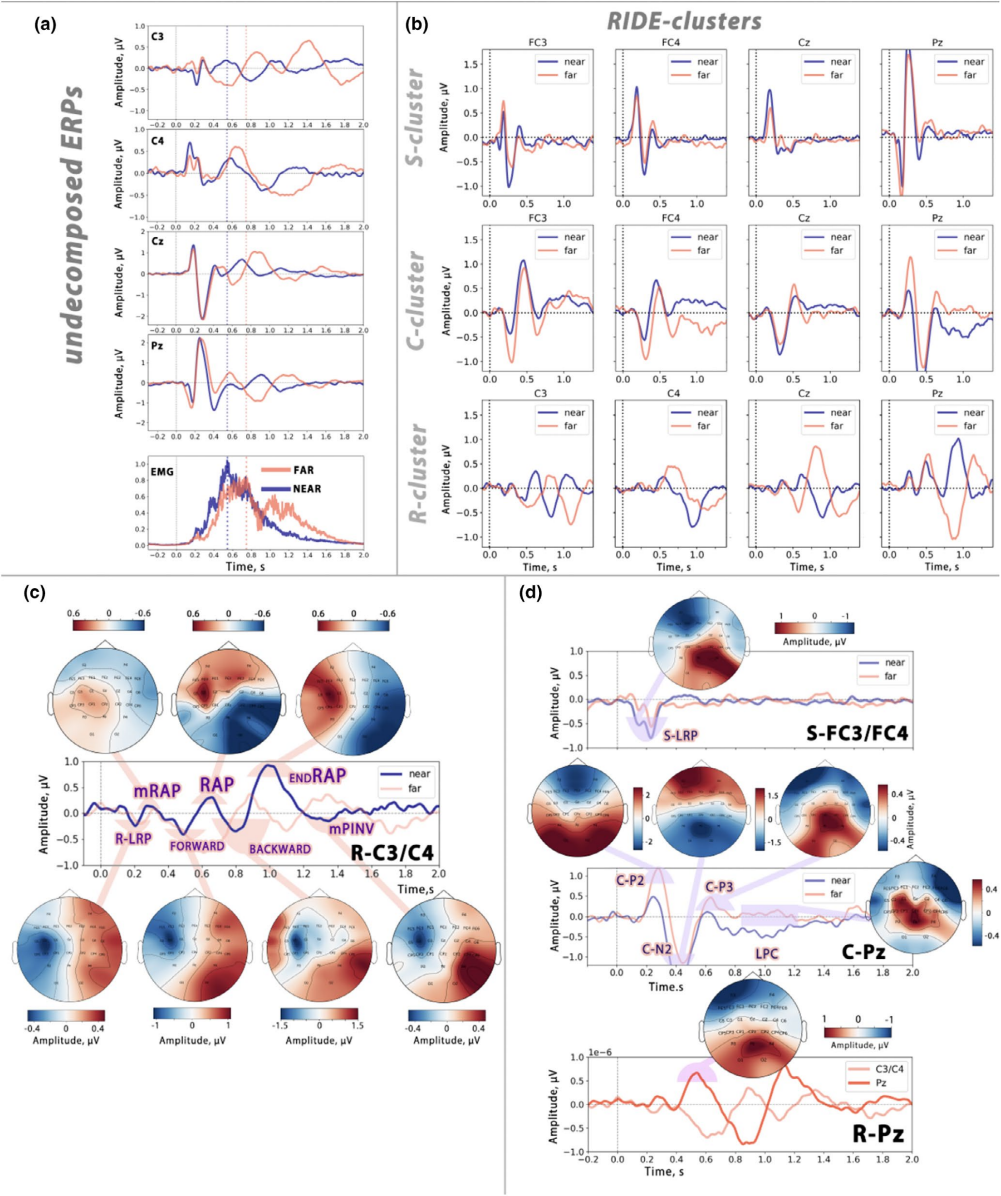
Waveforms of movement‐related cortical potentials and scalp topography maps in *far* and *near* trials. (a) Undecomposed stimulus‐locked grand mean evoked potentials in different channels. Averaged RMS‐transformed EMG responses in *far* and *near* responses are shown below. (b) Waveforms of RIDE clusters in different channels for far and near trials. (c) Lateralized R‐cluster activity obtained by subtracting *C4* signals from *C3*. Each motor response phase is represented by a sequence of negative and positive lateralized deflections. R‐LRP refers to the R‐cluster lateralized readiness potential, mRAP indicates lateralized activity derived from the RAP associated with muscle activation, forward and backward movements are characterized by slow negative contralateral waves, while RAP and endRAP indicate peaks associated with afferent processing during button press and response termination as the start button. Note that the late deflections for *far* and *near* trials are not synchronized. The scalp topography map for each peak response of the near condition is shown. (d) The top panel shows waveform and scalp topography of lateralized S‐cluster activity obtained by subtracting *C4* signals from *C3*. S‐LRP refers to the lateralized S‐cluster readiness potential. The middle panel shows C‐cluster activity from the *Pz* channel. Fronto‐parietal peaks (C‐P2, C‐N2, and C‐P3) and the late positive complex are shown with their topomaps indicating their fronto‐parietal scalp topographic distribution. The bottom panels show R‐cluster activity from the *Pz* channel. R‐P3 potential is shown with its parietal scalp distribution.

For visual inspection of MRCP components in undecomposed signals and comparison with RIDE results, all raw” epochs were averaged in a stimulus‐locked fashion to obtain evoked activity. Figure 3 shows the grand mean waveforms representing undecomposed MRCPs and clusters obtained by the RIDE procedure for both *far* and *near* conditions. In Figure 3a, it is evident that the cortical responses vary at different scalp positions for two movement distances, especially in the *C3* channel. Furthermore, these differences are more pronounced in the later phases, that is, just before or during movement execution. This observation suggests that response‐related neural activity plays an important role in generating these differences. It is worth noting that the EMG signal analysis shows the same movement onsets for both *far* and *near* trials. This finding further supports a high degree of similarity in the early, but not late, MRCP peaks in *far* and *near* conditions.

Indeed, the results of the RIDE, as shown in Figure 3b, indicate that the divergence between *far* and *near* movements is most pronounced in the R‐cluster. In contrast, the differences observed in the C and S‐clusters are limited to the amplitude of the peaks, rather than the latency. Specifically, within the R‐cluster, a distinct sequence of movement‐related events is evident, characterized by alternating negative and positive peaks. Figure 3c shows the R‐cluster data processed by subtracting the *C3* and *C4* signals. The initial small negative peak is the response locked‐lateralized readiness potential (R‐LRP), followed by a positive peak that coincides with the onset of EMG activity. This peak is interpreted as RAP related to muscle activation or mRAP (note that both R‐LRP and mRAP are congruent for both *far* and *near* conditions). Subsequently, a strong negative deflection is observed during the movement toward the button, transitioning into a second positive peak associated with the button press (RAP). The backward movement phase is also characterized by a negative wave, followed by a final positive peak, corresponding with the moment of termination of the movement at the start point (see endRAP peak in Figure 3c). In addition, Figure 3c clearly illustrates the contralateral predominance of these response‐locked peaks, confirming their association with activation of sensorimotor areas during movement.

Figure 3d shows the stimulus locked‐lateralized readiness potential (S‐LRP) derived by subtracting the *FC3/FC4* signals. These channels were chosen because the scalp distribution of the premovement negative peak in the S‐cluster was observed to have a more frontal appearance compared to the LRP in the R‐cluster. Notably, S‐LRP seems to be greater for *near* conditions, however, the permutation test did not find significance for this difference.

The C‐cluster (see Figure 3d) shows a distinct pattern characterized by a complex of three P3‐like fronto‐parietal potentials peaking at *FCz* and *Pz* (titled as С‐P2, С‐N2, and С‐P3). Although Figure 3d shows amplitude differences between the *far* and *near* conditions for these three peaks, a permutation test did not provide statistical significance for these observations. At the same time, we found a significant cluster falling in the late time range, approximately 700–1500 ms after stimulus, corresponding to LPC activity (Figure 6, see description below).

In the R‐cluster, we also observed a parietally distributed P3 peak (R‐P3) that appeared in the same latency range for both *far* and *near* conditions, and the amplitude for this peak also didn't differ between the two distances.

### 3.2 Behavioral results

**FIGURE 4:**
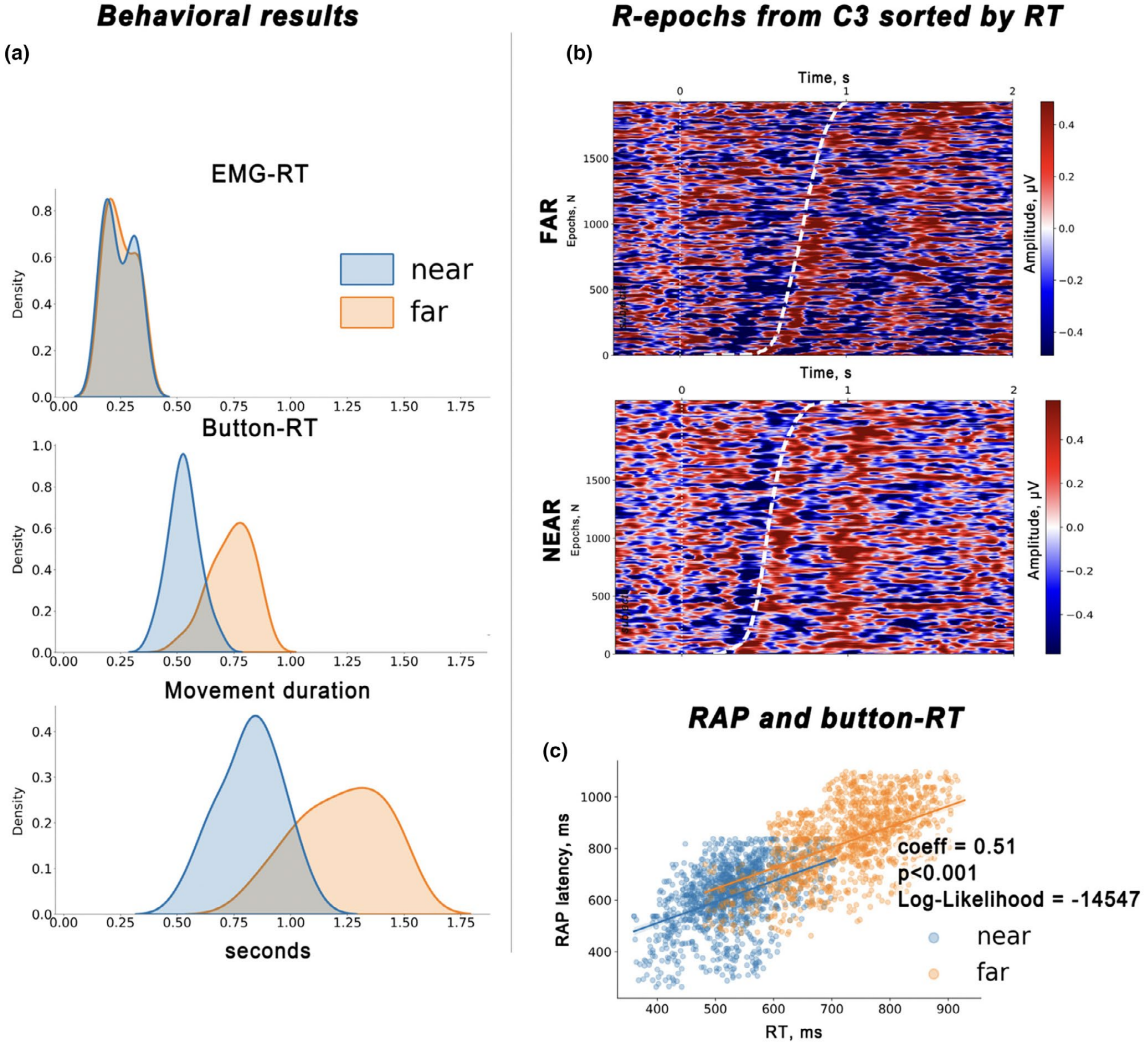
Relationships between R‐cluster responses and response times. (a) Behavioral metrics that characterize motor performance for the group of subjects: EMG‐RT refers to the reaction time calculated from the rise of the EMG signal indicating the start of the movement; Button‐RT refers to the moment of pressing the target button (far or near); movement duration was obtained by subtracting EMG‐RT from Button‐RT in each trial. (b) Single‐trial R‐signals from channel *C3* are plotted in ascending response time (RT) order, with a bold dashed white line marking the RT. Note the appearance of the RAP peak immediately after the button is pressed. (c) Graph shows the correlation between RAP peak latency and RT, where each point represents a single trial. The results of the LMM analysis are shown in the legend.

This further analysis focused on correlating cortical activations in R‐ and C‐clusters with behavioral outcomes. Figure 4a shows the group distribution of RTs obtained from EMG and keypress measurements. The EMG RT, defined as the moment of movement onset determined by the rise of the EMG signal, shows significant overlap for *near* and *far* trials. This indicates that the movement started with the same delay for both targets. This is supported by the equal latency of R‐LRP and mRAP in Figure 3c In contrast, button RT, defined as the moment the button was pressed, shows a clear distinction between the *far* and *near* conditions, indicating that it takes longer to reach and press the *far* target. Accordingly, for the *far* button, the movement time was longer.

### 3.3 R‐cluster and button pressing time

To comprehensively determine the relationships between RAP and button press reafference, we sorted all R‐cluster trials from all subjects by button press delay in ascending order. Figure 4b shows the result of sorting for both distances separately, and clearly shows that the RAP appears just after the button press event. We also statistically examined the trial‐level relationship between RAP latency and button RT using linear mixed models. A significant positive effect of RT delay on RAP latency was found (*estimated coefficient* = 0.51, *p* < 0.001, *standard error* = 0.04, *z* = 11.55, *Log‐Likelihood* = −14.547, see Figure 4c).

### 3.4 C‐cluster and movement duration

**FIGURE 5:**
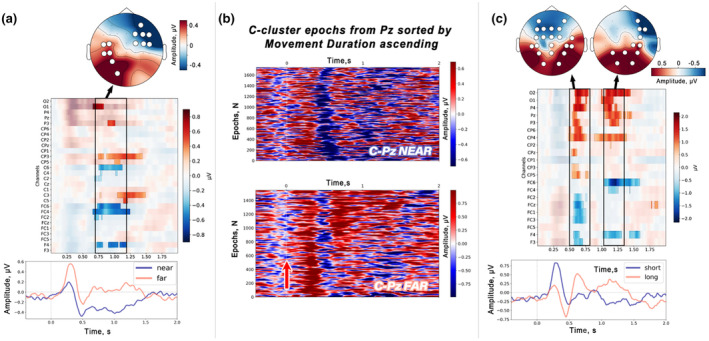
C‐cluster signal differences in *far* vs. *near* trials and motion duration effects. (a) Permutation statistical test results show *far* and *near* response differences within an LPC‐aligned interval. A color panel shows C‐cluster amplitudes across channels, with a mask highlighting where significant distance differences were found. The topography map shows the scalp distribution of signal amplitudes in this interval, with channels included in significant clusters highlighted. (b) Single‐trial C‐signals from the *Pz* channel for both conditions, ordered by increasing movement duration. The red arrow indicates the direction of movement duration increase. (c) Permutation test results show significant clusters discriminating short from long movements in the *far* condition. Temporal characteristics of the clusters indicate differences within the LPC activity interval, spatial characteristics indicate the fronto‐parietal channels included in the cluster (topographic maps are provided).

We observed significant differences between the *far* and *near* conditions within the C‐cluster signals. This corresponded to a cluster in the observed data beginning approximately 700 ms after the stimulus, within the latency range of late latency positivities (cluster *p*‐values < 0.001). The significant cluster included fronto‐parietal channels, as can be seen in Figure 5a.

Given that the C‐cluster is thought to reflect correlates of movement monitoring, we proposed that its shape might vary within a condition with movement duration. To explore this idea, we computed the movement duration separately for the *far* and *near* conditions by calculating the interval from EMG onset to button RT in each trial. We then selected trials corresponding to the first and last quartiles of the movement duration distribution for each participant, thereby isolating C‐cluster signals associated with the longest and shortest movements across conditions. Comparison of the longest and shortest movements revealed differences in the C‐cluster only in the *far* target condition. A permutation test identified three spatiotemporal clusters (*p* < 0.001). These clusters correspond to time intervals with three P3‐like fronto‐parietal potentials and LPC activity. This activity was more pronounced in trials with longer movements and falls approximately in the 1000–1500 ms latency range. Figure 5b,c illustrates the result of sorting the C‐cluster trials based on ascending movement duration for the *far* and *near* conditions, as well as the results of the spatiotemporal permutation analysis.

### 3.5 Multivariate pattern analysis results

**FIGURE 6:**
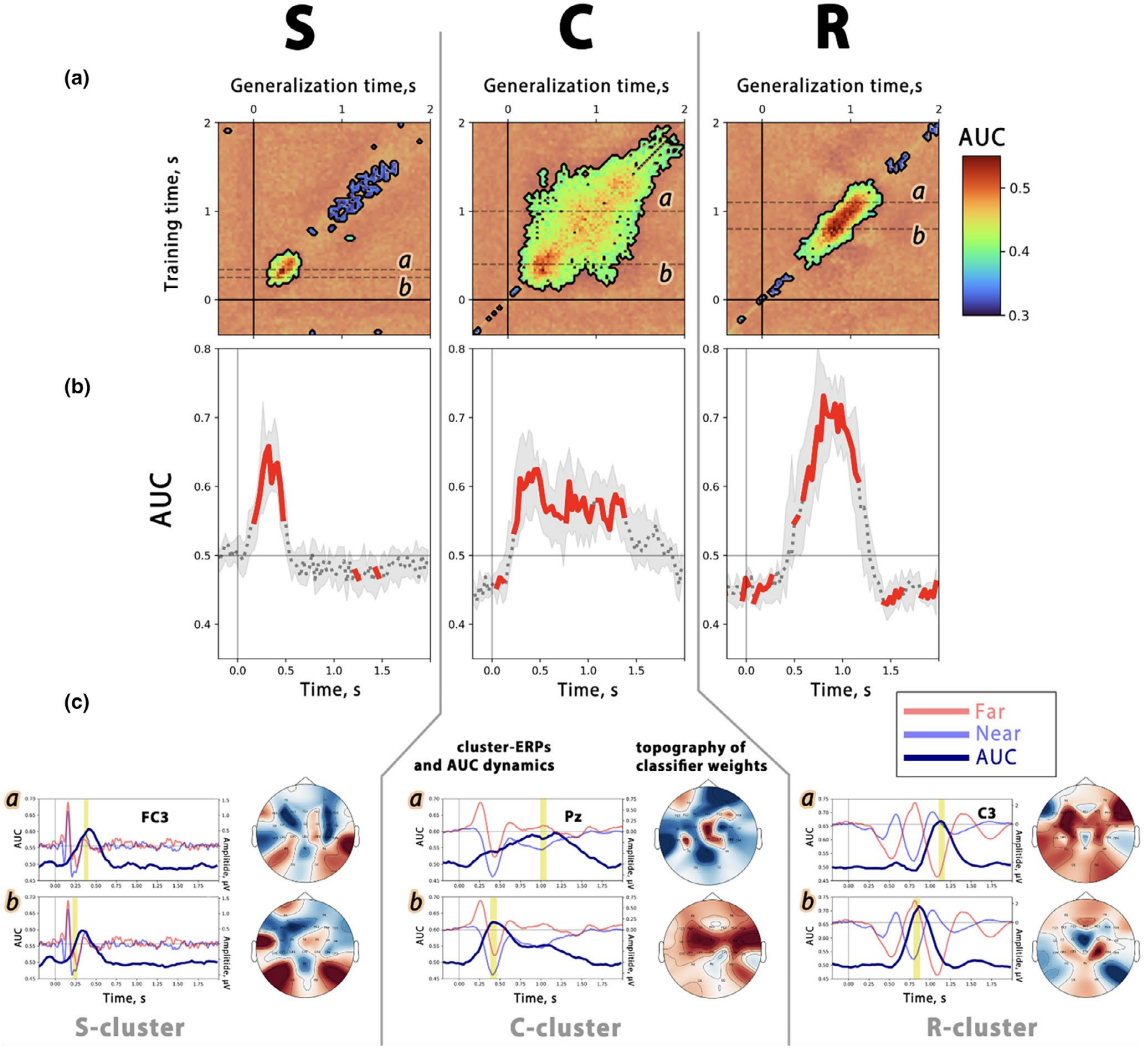
MVPA results. (a) Temporal generalization matrices separately for the RIDE decomposed C‐, R‐, and S‐clusters. The plots show how a classifier trained at specific points in time generalizes across the trial timeline. The performance of the classifier is shown on a scale, with diagonal lines indicating the effectiveness of training and testing at the same time. Masks highlight areas of statistically significant decoding (*p* < 0.01; 2‐sided cluster‐based permutation). Notably, the S‐cluster shows brief diagonal activation between 150 and 550 ms, the C‐cluster shows extended diagonal and off‐diagonal activity from 100 ms to the end of the trial, and the R‐cluster shows focused diagonal activation from 500 to 1200 ms. (b) Classification performance between far and near trials, represented as AUC separately for the RIDE‐decomposed C‐, R‐, and S‐clusters (diagonal values from the presentation of the above temporal generalization matrices). Time 0 indicates the presentation of the visual stimulus. Bold red lines indicate significant time windows (*p* < 0.01; 2‐sided cluster‐based permutation). (c) Decoding as a function of time. These curves represent the AUC values obtained from classifiers trained at specific time points (labeled *a* and *b*) and tested over the entire epoch. The topographical maps show the classification coefficients, which indicate the weight of each channel. Topographic maps show channels with the most significant impact on classification performance. Yellow vertical areas indicate time intervals corresponding to the topographic maps.

Figure 6 shows the results of the multivariate pattern analysis. Section (A) shows the temporal generalization matrices that illustrate the temporal stability of classification performance between *far* and *near* conditions for the three RIDE clusters. Section (B) shows decoding accuracy dynamics in terms of AUC values from the main diagonal of the matrices Within the S‐cluster, the generalization matrix showed a short activation in the 150–550 ms window after stimulus presentation (classification was significantly above chance within this interval, see Figure 6b). Conversely, the C‐cluster showed a sustained diagonal activation from 100 ms to the end of the trial. The extensive off‐diagonal activation pattern observed in the C‐cluster matrix indicates a temporally sustained differentiation in information encoding within this cluster, effectively distinguishing between *far* and *near* movements. In the R‐cluster, a narrower activation was observed, suggesting that response‐related activity differentiates between *near* and *far* conditions within the 500–1200 ms interval after the visual cue. Therefore, the MVPA classification results of the temporally decomposed MRPCs revealed that target distance modulates brain activity in all *object*, *action*, and *event* *files*.

To explicitly determine which ERP components were pivotal in distinguishing between the two movement conditions, we employed generalization curves (see Figure 6c). These curves represent the AUC values, obtained from classifiers trained at specific time points (labeled with *a* and *b* letters in Figure 6a) and tested across the entire epoch. The classifiers' coefficients were visualized as topographical maps to identify the channels with the most significant impact on classification performance. Our analysis revealed that the S‐LRP and its subsequent positive peak were crucial in differentiating the S‐cluster signals between the *far* and *near* conditions. The fronto‐central channels, especially those from the contralateral hemisphere in the case of the S‐LRP, showed higher weights in the fitted models. For the C‐cluster, the key activity discriminating between *far* and *near* conditions included all three P3‐like peaks and the subsequent slow LPC. As expected, the R‐cluster was the most successful at discriminating between *far* and *near* movements (it exhibited the greatest AUC score across all clusters). The highest AUC was found at points where the *far* and *near* R‐cluster signals in two conditions showed oppositely directed peaks. Notably, the channels with greater influence in the classification were predominantly located over the fronto‐central areas.

## 4 DISCUSSION

In the current study, we used an SRT paradigm with two buttons linearly spaced at different distances from the participant. Participants were instructed to reach for and press either the *far* or *near* button in response to a visual stimulus. This allowed us to dissociate the processes of S–R mapping and muscle activation from the activity associated with subsequent reaching and movement outcome assessment. To disentangle the temporal sequence of motor processes as well as parallel processes such as action monitoring and context updating, we used the RIDE algorithm. This approach allowed us to decompose MRCPs into three distinct clusters, each representing fundamental processes from perception to action execution.

### 4.1 Sequence of peaks associated with movement and afferentation processing in R‐cluster

Undecomposed evoked potentials were characterized by peaks with distinctly different latencies between *far* and *near* trials (Figure 3a). We believe that these components are directly related to response execution, as confirmed by the decomposition procedure. After RIDE decomposition, only the R‐cluster showed an alternating pattern of negative and positive deflections, forming a movement‐evoked “oscillatory” pattern, particularly evident in *C3* signals and lateralized MRCPs (Figure 3). Based on their scalp distribution, these deflections can be identified as components related to sensorimotor activation stages during motor execution and sensory feedback processing. The alternating peaks in the R‐cluster indicate that feature code activation in the *action file* occurs at each stage of the movement, in a consecutive manner. The following sections will describe each component and its possible nature.

#### 4.1.1 Lateralized readiness potential

The first lateralized negative deflection, appearing before the EMG rise, is R‐LRP, reflecting response‐locked primary motor cortex activation. Premotor lateralization was also observed in the S‐cluster. In SRT paradigms, visuomotor transformation involves two key processes: visuomotor priming (perceptual stage) and response selection (low‐level motor process) (Hackley & Valle‐Inclan, 1998; Stürmer et al., 2013). Both contribute to LRP formation and can be studied separately via stimulus‐ and response‐locked averaging (Berchicci et al., 2016). Using RIDE, our study, along with those by Stürmer et al. (2013) and Takacs et al., 2020, Takacs et al., 2021a, differentiated S‐LRP and R‐LRP. S‐LRP indicates the transition from stimulus processing to response selection, while R‐LRP represents the final preparatory motor stage (Rinkenauer et al., 2004). For both R‐ and S‐LRPs, we found no significant differences between the two target distances, contrasting with literature suggesting that LRP amplitude correlates positively with the muscle effort required for movement execution (Kirsch et al., 2010). At the same time, our results are consistent with a study (Kourtis et al., 2012) using a similar methodology to ours, which showed that distance to the target did not affect the LRP, but it significantly affected the amplitude of the N2 and P3 components (Kourtis et al., 2012).

#### 4.1.2 Reafferent potentials

After the R‐LRP, a small positive peak appeared in the R‐cluster signals, which we interpret as the RAP associated with the processing of muscle activation‐induced afferents (mRAP in Figure 3c) (Di Russo et al., 2005; Kornhuber & Deecke, 1965; Rauchbauer et al., 2018; Shibasaki et al., 1980). As has been previously demonstrated in numerous studies, the RAP is identified as a positive peak that develops in succession with the LRP and immediately following the pressing of the key (Syrov et al., 2024). However, a majority of previous studies employed SRT tasks with a minimal interval between muscle activation and key press (Di Russo et al., 2005; Syrov et al., 2024) or even employed simple movements without button pressing (Kornhuber & Deecke, 1965; Rauchbauer et al., 2018). This limitation has precluded the clear delineation of whether the RAP is specifically associated with the processing of movement goal attainment or if it serves as a general neural marker of sensory processing, such as muscle and skin sensations. In our study, we were able to clearly identify the mRAP peak associated with both kinesthetic and tactile afferency that appeared immediately after the onset of the EMG rise. At the same time, we detected a second, larger positive peak after the button press with a similar contralateral scalp distribution as mRAP (see RAP in Figure 3c). We attribute this latter RAP to the processing of afferent signals induced by the button press. We also acknowledge the potential contribution of muscle contractions associated with backward movement to the formation of this RAP peak. To explore the relationship between this second RAP and button RT, we performed a sorting analysis of R‐cluster trials based on their respective RTs. The RAP was clearly aligned with the RT line in both conditions, appearing approximately 50 ms after both presses. This relationship was also confirmed with LMM analysis, which revealed a significant positive effect of RT delay on RAP latency.

In both conditions, we also observed positive contralateral peaks coinciding with movement termination (1000–1100 ms for near and 1300–1400 ms for *far* trials, as shown in Figure 3a–c). This peak coincides with the moment when the hand returns to the starting point, which leads us to classify it as a RAP related to the *start* button press (see endRAP in Figure 3c).

#### 4.1.3 Execution‐related negative potentials

The moments of reaching toward and returning from the target buttons were characterized by pronounced negative contralateral potentials. These potentials were more prolonged and larger in amplitude in *far* trials. Although a few studies have explicitly discussed such activity during reaching movements, hypotheses about its nature are controversial. Some studies attribute these negative peaks to movement‐related feedback processing during execution (Deecke et al., 1976; Shibasaki et al., 1980; Bender et al., 2006). In contrast to this interpretation, we rather support Kirsch et al. (2010), who described a similar execution‐related negativity as the motor N400. This was hypothesized to be distance‐specific brain activity correlated with the acceleration and deceleration phases of goal‐directed hand movements (Bender et al., 2006; Kirsch et al., 2010). Several other studies have also found an association between motor N400 and movement kinematics in reaching and grasping movements (Koester et al., 2016; Westerholz et al., 2013). Thus, the execution‐related negativity may be indicative of the control of muscle force during reaching, analogous to the LRP, which was postulated to be associated with the modulation of the initial force impulse (Semionow et al., 2000; Slobounov et al., 1999, 2000).

#### 4.1.4 Motor post‐imperative negative variation

Following endRAP, a slow negative deflection was observed (see mPINV in Figure 3c). This deflection peaked in the *C3* channel at approximately 1300 ms after the stimulus for *near* trials and around 1800 ms for *far* trials. The similar delayed contralateral activity was described by Bender et al., 2006 as the mPINV, suggesting that reafferent sensations or muscle activation do not sufficiently explain its origin. The well‐studied *classical* PINV is known to integrate both cognitive and motor components (Verleger et al., 1999; Werner et al., 2011; Bender et al., 2012). Its amplitude has been found to correlate with uncertainty about the correctness of performance (Werner et al., 2011). Bender et al. (2006) described mPINV as a motor component associated with action outcome estimation and identified its origin in the pre‐/primary motor cortex. This highlights the role of these areas in evaluating the success of a movement. Consequently, mPINV can be regarded as a marker for the post‐movement validation of actual outcomes by comparing them with internal movement representations (Thiemann et al., 2012). The results of our study, indicating that mPINV is attributed to the R‐cluster, provide evidence for the inclusion of processes related to post‐movement outcome validation in the *action* file.

### 4.2 Action monitoring and C‐cluster

The C‐cluster is believed to contain signals with variable latency across trials. Previous research has identified the role of the C‐cluster in *event file* encoding, including S–R mapping, task rule updating, and response monitoring (Kleimaker et al., 2020; Opitz et al., 2020; Takacs, Mückschel et al., 2020; Eggert et al., 2022). This information, pertaining to the representation of S–R features, is retrieved from short‐term memory in response to a visual stimulus, thereby enabling the execution and control of the corresponding motor action (Hommel & Frings, 2020; Kühn et al., 2011). The neural substrate of the C‐cluster is based on the fronto‐parietal networks, which are involved in the parallel processing of S–R integration for response preparation and monitoring, that is, *event file* attributes (Brydges & Barceló, 2018; Naranjo et al., 2007; Thoenissen et al., 2002).

The results of our analysis indicate that the C‐cluster contains multiple components with a fronto‐parietal scalp distribution. We have identified the first three components as P3‐like peaks (see P2, N2, P3 in Figure 3c), which are followed by a complex of late parietal positive peaks. Previous EEG studies employing SRT have found these specific components to play a crucial role in perception‐action coupling and context updating (Brydges & Barceló, 2018; Kourtis et al., 2012; Kleimaker et al., 2020; Takacs et al., 2021a; Verleger et al., 2014, 2016, 2020).

The study by Kourtis et al. (2012) highlighted the effect of movement distance on the amplitude of premovement fronto‐parietal N2 and P3 components and discussed their role in transmitting information about movement difficulty to the motor programming level. In contrast to their findings, our study did not observe statistical differences in C‐cluster signals within early fronto‐parietal peaks. However, we did find differences between *far* and *near* movements within late‐latency positive components (~600–1500 ms post‐stimulus). This period corresponds to the backward movement in the *near* condition, whereas in the *far* trials participants were still reaching for the target. Despite uncertainty about its nature, the LPC, also known as the positive slow wave (Dien et al., 2004; Goldstein et al., 2002), is thought to be functionally similar to the P3 family. We compare its role in our conditions to the late parietal P3 described by Verleger et al. (2005). This activity integrates perception and response and underlies a monitoring process that ensures that initial decisions and subsequent actions are appropriately aligned. In our study, however, LPC exhibited a significantly greater delay compared to the findings of Verleger et al. (2005). We attribute this discrepancy to the prolonged duration of reaching movements in our trials, which necessitated longer monitoring until the completion of the action. This hypothesis is supported by the observed differences in C‐P3 and LPC between short‐ and long‐duration movements within the *far* condition (Courchesne, 1978). Importantly, the separation of *far* trials based on movement duration contributed to a more pronounced dissociation between the C‐P3 and LPC components in long‐duration trials (see Figure 5). This observation is consistent with the existing literature, which identifies the P3 and adjacent peaks as part of an ensemble involved in detecting deviations from the internal model and alerting the central executive for further evaluation of events (Brydges & Barceló, 2018; Dien et al., 2004).

Consequently, our findings contribute to the hypothesis that C‐cluster signals reflect neural activity associated with continuous action monitoring (Blakemore & Sirigu, 2003; Takács et al., 2022; Eggert et al., 2022) and support the notion of the TEC, which posits that activity in the *event file* is continuously updated, allowing for necessary adjustments (Hommel, 2009).

### 4.3 Response‐related P3

Interestingly, we also found a parietal P3 peak in the R‐cluster. This peak, although attributed to response‐related signals, did not show latency differences between *far* and *near* trials. A parietally distributed R‐P3 positivity was observed in (Brydges & Barceló, 2018), where it was associated with the updating of (pre‐)motor units of sensorimotor S–R mappings in a task‐switching paradigm. Previous research on response‐locked P3‐like activity is limited. However, Gajewski and Falkenstein (2011) and Verleger et al. (2016) identified similar R‐P3 activity that occurs 40–80 ms before the response. Consistent with Verleger et al. (2016), we found that R‐P3 is not related to button press time, and our results actually enrich Verleger's findings showing independence of R‐P3 latency from movement distance. We find it interesting that R‐P3 does not show distance‐related latency shifts, but has been attributed to the R‐cluster.

### 4.4 Multivariate pattern analysis results

Going beyond traditional time‐domain analysis, we implemented multivariate pattern analysis to delineate neural representation differences between *far* and *near* conditions. This advanced approach, which is sensitive to the temporal consistency of neural patterns (Fahrenfort et al., 2018; Grootswagers et al., 2017; King & Dehaene, 2014), allowed us to unravel distance‐related signatures in *action*, *object*, and *event* *files* (King & Dehaene, 2014). Our results show a stable, diagonal pattern in the R‐cluster, that is, disparities in action feature codes for *far* and *near* trials that appear ~500 ms after the stimulus, where actions in the two conditions diverge: the near button is already pressed, whereas in far trials participants are still reaching. In contrast, the S‐cluster signals showed significant decoding accuracy at early stages, exactly within the temporal window of the S‐LRP. This suggests that distance‐related properties are manifested at the perceptual level, which is in line with the literature indicating stimulus‐locked LRP sensitivity to target distance (Kirsch et al., 2010). The lack of differences in the S‐LRP between the two conditions in the permutation tests, in particular, highlights the sensitivity of MVPA to the detection of subtle neural distinctions (Li et al., 2022; Takacs et al., 2020).

The prolonged off‐diagonal activity observed in the C‐cluster signals the long‐lasting maintenance of corresponding neural representations. This persistent pattern occurs prior to movement onset, suggesting distance‐specific activation during preparatory stages. This is consistent with the TEC, which proposes that processing of target distance within the *event* *file* begins during S–R mapping and continues with a sustained activity until the end of the trial, when the response is achieved and the movement is terminated (Hommel et al., 2001).

However, we would like to note that the long‐lasting decoding observed in the C‐cluster, similar to the results of the R‐cluster, is due to the shorter activation in the *near* trials. After about 500 ms, when the *near* button was pressed, the responses in the two conditions diverged significantly. However, our focus here is primarily on the off‐diagonal activation in the C‐cluster, rather than the difference between *far* and *near* trials. This pattern indicates the persistence of stable C‐related activity throughout execution, in contrast to the R‐cluster where different phases alternated. This persistent activation has been replicated in several studies (King & Dehaene, 2014; Takacs et al., 2020; Vékony et al., 2023), confirming its integral function throughout the response execution continuum.

## 5 CONCLUSION

The present study focused on the components of MRCPs related to movement preparation, execution, and monitoring. The application of a signal decomposition technique enabled the description of neural signatures reflective of response monitoring and outcome assessment at different levels.

In contrast to traditional views that exclusively attribute response monitoring to the *event* *file*, our results indicate that the *action* *file* also contains signals associated with response outcome control. Specifically, we identified mPINV in response‐related cluster and several RAP peaks associated with key stages of the response, namely, muscle contraction in movement initiation and target button press. The utilization of two distinct target distances enabled the differentiation of these components, which are typically intermixed during short transient movements. These findings indicate the significance of sensorimotor afferent processing at pivotal moments of an action.

Furthermore, we examined C‐clusters containing *event* *file* signals, which are associated with S–R mapping and high‐level response monitoring. We described several subsequent fronto‐parietal components in this cluster, demonstrating the activation of the *event* *file* from the early preparatory stages, through the achievement of response goals, to termination. It is notable that late parietal positivity, which exhibited sustained activity throughout the execution process, was sensitive to movement duration.

## 6 LIMITATIONS

One limitation of our analysis is the use of single subtraction (*C4* − *C3*) instead of the traditional double subtraction method proposed by Coles (1989). This might reduce the reliability of estimating motor‐related lateralized activity. Concurrently, single subtraction has its own advantages and allows the analysis of lateralized components within single trials, as well as the exploration of relationships between lateralized potentials and behavioral variability (*e.g*., RT), as was utilized in Falkenstein et al. (2006) and Syrov et al. (2024).

To further strengthen our results, we used the RIDE technique, which separates motor‐related components from other activities, ensuring that lateralization in the R‐cluster was specific to motor activity. While S‐LRP could still be contaminated by non‐motor asymmetries, this is unlikely because stimuli were not presented laterally in the visual field.

Although handedness affects LRP amplitude (Schmitz et al., 2019) and results might vary without double subtraction, all subjects in our study were right‐handed, which we believe ensures uniformity. However, we did not assess the degree of right‐hand dominance, so our results should be interpreted with this limitation in mind.

## AUTHOR CONTRIBUTIONS

NS and DGM equally contributed to the conception and design of the experiments; data collection and analysis; results interpretation; drafting of the manuscript; editing and revision of the manuscript. AM contributed to data collection; results interpretation and drafting of the manuscript. LY contributed to the results interpretation; editing and revision of the manuscript. AK and ML supervised the study.

## FUNDING INFORMATION

Russian Science Foundation, grant #21‐75‐30024.

## CONFLICT OF INTEREST STATEMENT

The authors have no conflicts of interest to declare that are relevant to the content of this article.

## Supporting information


Appendix S1.

## Data Availability

The data used in this study were uploaded to the open science framework (OSF) platform, and it is accessible through https://osf.io/kvsrb/. The Python script used for the preprocessing of the data and the application of MVPA is available based on the request to the authors.
