# Supplementary material for: Revealing the different levels of action monitoring in visuomotor transformation task: Evidence from decomposition of cortical potentials
Source: Psychophysiology. 2024 Oct 14;62(1):e14708. doi: 10.1111/psyp.14708 (PMC11785542; doi:10.1111/psyp.14708)

Appendix 1: EMG-RT and Button press-RT RIDE decomposition

The figure shows the comparison of RIDE decomposition using reaction time calculated from EMG RT and button press time. The EMG RT was defined as the time when the root mean square (RMS) transformed EMG signal exceeded two standard deviations above the baseline level. The button-press RT was obtained as the time when the target button was pressed. As can be seen in the figure, the decomposition using the button-press RT yielded a more prominent R-cluster, suggesting a more reliable separation of C- and R-clusters.

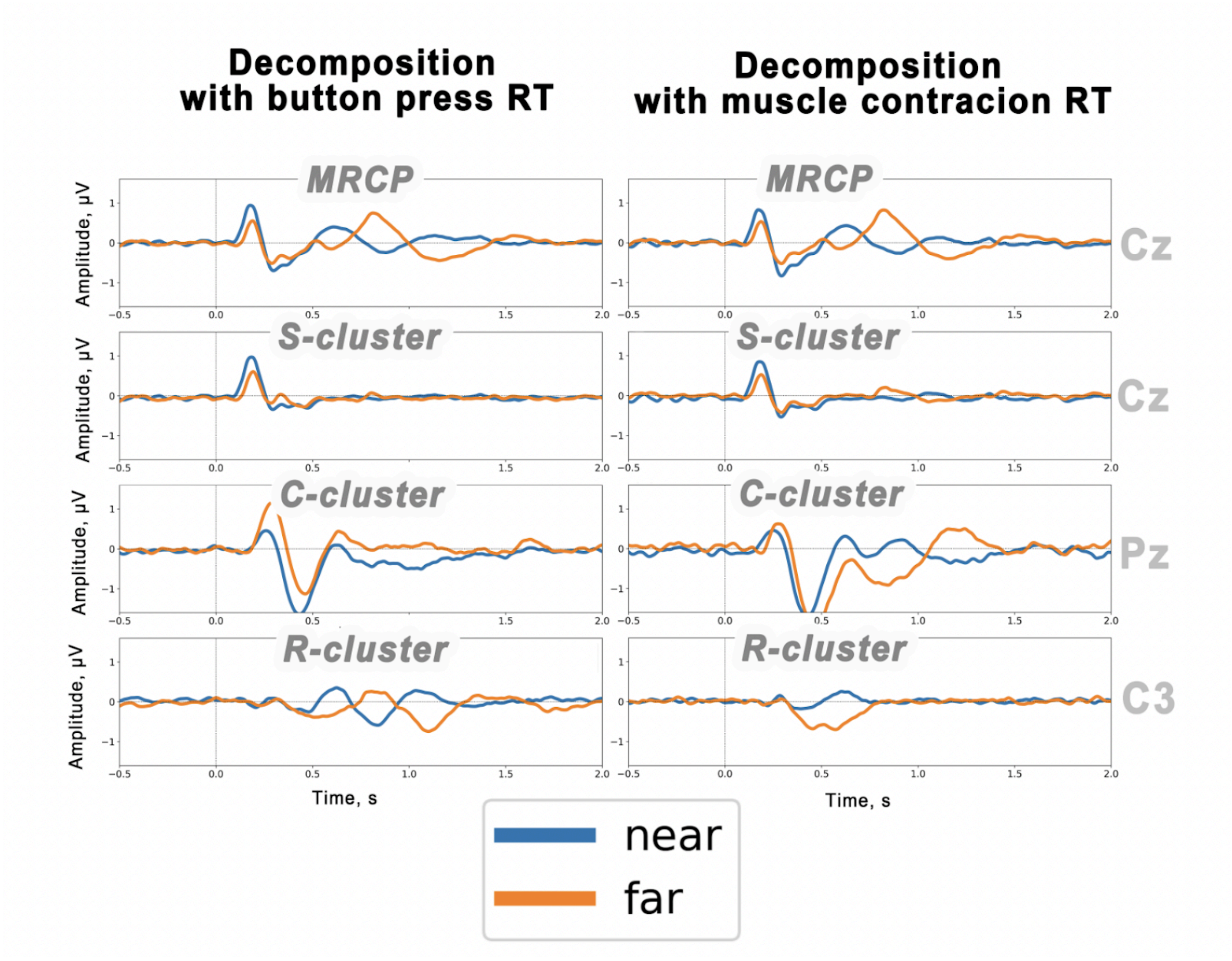

Supplement: Supplementary file 1 — Appendix S1. [file PSYP-62-e14708-s001.pdf]
